# Local Application of BMP-2 Specific Plasmids in Fibrin Glue does not Promote Implant Fixation

**DOI:** 10.1186/1471-2474-12-163

**Published:** 2011-07-15

**Authors:** Benjamin Faensen, Britt Wildemann, Christian Hain, Julius Höhne, Yvonne Funke, Christan Plank, Axel Stemberger, Gerhard Schmidmaier

**Affiliations:** 1 Center for Musculoskeletal Surgery, Charité - Universitätsmedizin Berlin, Germany; 2 Julius Wolff Institute and Berlin-Brandenburg Center for Regenerative Therapies, Charité - Universitätsmedizin Berlin, Germany; 3 Institute of Experimental Oncology, TU München, Germany; 4 Department für Orthopädie, Unfallchirurgie und Paraplegiologie, Universitätsklinik Heidelberg, Germany

**Keywords:** BMP-2, gene transfer, non viral gene vector, COPROG, implant healing, fibrin glue

## Abstract

**Background:**

BMP-2 is known to accelerate fracture healing and might also enhance osseointegration and implant fixation. Application of recombinant BMP-2 has a time-limited effect. Therefore, a gene transfer approach with a steady production of BMP-2 appears to be attractive. The aim of this study was to examine the effect of locally applied BMP-2 plasmids on the bone-implant integration in a non-weight bearing rabbit tibia model using a comparatively new non-viral copolymer-protected gene vector (COPROG).

**Methods:**

Sixty rabbits were divided into 4 groups. All of them received nailing of both tibiae. The verum group had the nails inserted with the COPROG vector and BMP-2 plasmids using fibrin glue as a carrier. Controls were a group with fibrin glue only and a blank group. After 28 and 56 days, these three groups were sacrificed and one tibia was randomly chosen for biomechanical testing, while the other tibia underwent histomorphometrical examination. In a fourth group, a reporter-gene was incorporated in the fibrin glue instead of the BMP-2 formula to prove that transfection was successful.

**Results:**

Implant fixation strength was significantly lower after 28 and 56 days in the verum group. Histomorphometry supported the findings after 28 days, showing less bone-implant contact.

In the fourth group, successful transfection could be confirmed by detection of the reporter-gene in 20 of 22 tibiae. But, also systemic reporter-gene expression was found in heterotopic locations, showing an undesired spreading of the locally applied gene formula.

**Conclusion:**

Our results underline the transfecting capability of this vector and support the idea that BMP-2 might diminish osseointegration. Further studies are necessary to specify the exact mechanisms and the systemic effects.

## Background

Total hip and total knee arthroplasties (THA, TKA) in the industrialized countries are increasing and demographic data suggest that this progress is going to continue [1-3]. According to the Swedish National Total Hip Arthroplasty Register with more than 270,000 registered THA from 1979-2006, the leading cause for component failure in THA is aseptic implant loosening [4]. Besides osteolysis due to wear debris (especially of polyethylene components) it is assumed that a lack of initial bony incorporation of the implant favors aseptic loosening. Bone ingrowth does not occur properly if micromotion exceeds 150 μm [5]. Therefore, many attempts have been made to improve the incorporation of the implant. The design of the prosthesis has a large impact on primary stability. Modifications to the implant surface, such as different micro- and macrostructures or osteoconductive coatings (e.g. hydroxylapatite), have shown to play a decisive role in improving primary as well as secondary stability, due to bone ingrowth [6-8].

It is well accepted that certain growth factors (GF), mostly members of the transforming growth-factor superfamily, i.e. bone morphogenetic proteins (BMP) and transforming growth factor β (TGF-β) promote bone formation [9-11]. BMP-2 is well known to have high osteoinductive potency and to improve bone healing. In the last years, clinical application of BMPs has become common in the treatment of atrophic non-unions of shaft-fractures, open tibial fractures and spine-fusions[10]. In experimental studies also the improvement of implant incorporation into bone has been shown under the influence of BMP-2 [12,13] as well as of BMP-7 [14,15].

Regardless of the indication, providing a steady long-term delivery of recombinant growth factors to the site of action remains an unsolved problem. Therefore, the idea of a gene transfer system to establish a constant long-term but still temporally controlled local level of GF at the wound site appears to be an attractive method.

Gene therapy aims at the replacement of a defective or missing gene or at the additional insertion of an existing gene to start or stimulate the production of a certain gene product, e.g. a growth factor.

A vector is needed to insert the gene into a target cell. This vector is either of viral origin or it is a so called non-viral vector. In the latter group, a variety of techniques are used, including synthetic molecules or physical methods. A vector should have properties which enable it to carry the gene to the target cell and to invade the cell. Non-viral vectors generally show a relatively poor potency in introducing nucleic acids into cells *(transfection) *compared to viral vectors, where the introduction of nucleic acids into cells (*transduction) *is part of the natural viral life cycle. On the other hand, non-viral vectors are accepted to be safer than their potentially mutagenic or immunogenic viral counterparts [16,17].

The aim of this in vivo study was to investigate the influence of a plasmid encoding BMP-2 on implant incorporation in a non-weight bearing rabbit model. In addition we studied the effectiveness of the promising, comparatively new (non-viral) copolymer-protected gene vector (COPROG) [18,19] as well as safety aspects.

## Methods

All animal studies were approved by the proper authorities (Landesamt für Arbeitsschutz, Gesundheitsschutz und technische Sicherheit Berlin, Germany).

Sixty male New Zealand White rabbits (Harlaan-Winkelmann, Germany) with an average age of 8 months underwent surgery.

As a carrier for the vector formula, we used commercially available two component fibrin glue. Once bonded, the glue would keep the drug formula at the wound site. It has been proven that the incorporation of the plasmid formula does not change the properties of the glue, so that in can be applied as intended by the manufacturer [20].

An established animal model was chosen for the in vivo experiments. The rabbit tibia allows an easy surgical approach and the possibility to transfect bone tissue in New Zealand White Rabbits has already been shown before [21].

### Groups

The animals were divided in 4 groups. All animals received anterograde intramedullary titanium nails (2.5 mm diameter) in both tibiae. A common two-component fibrin sealant (Tissucol^®^, Baxter, Germany) was used as drug carrier, which was injected into the reamed tibia (2.8 mm) before inserting the nail. The fibrinogen component carried the gene vector.

There were three groups of 16 animals each and one group of 12 animals: 1. a control group which received the nail only, 2. a second control group (fibrin glue group) which received the nail with fibrin glue but without plasmids, and the 3. the verum group, received the nail with fibrin sealant and the plasmids. Half of the animals of each group were sacrificed after 28 days and the other half after 56 days. The tibiae of each animal were randomly assigned for either histomorphometry or biomechanics. In the fourth group, 12 animals served as the reporter-gene control group and the animals were sacrificed at 4, 7 and 28 days. Luciferase, an enzyme normally only expressed by the firefly, was used as the reporter-gene.

### Non viral vector

The non viral vector used in this study is a ***Co**polymer **Pro**tected **G**ene Vector (COPROG)*. It consists of a positively charged polycation-Plasmid DNA polyplex coated by a ***pro**tective anionic peptide-PEG **cop**olymer *(PROCOP), which diminishes the susceptibility of the complex to aggregation, to complement activation and interaction with serum proteases [18]. The non viral vector, provided in lyophilized form can easily be incorporated in the fibrin-component of the fibrin glue. In the verum group, it carried 84 micrograms of a plasmid encoding for human BMP-2 (pB-BMP-2). In the Luciferase group, the glue was carrying a plasmid encoding for the reporter-gene Luciferase (pCMV-luc). The plasmids were also provided by the Institute of Experimental Oncology, TU München, Germany. The amount of 84 micrograms of plasmid/implant is based on the manufacturing procedure of the plasmid/COPROG-mixture and represents the highest possible "load" of COPROG to the fibrin component without compromising the maximum clotting firmness (MCF) of the fibrin glue.

### Surgical procedure

After the animals were anaesthetized with Ketamine (90 mg/kg body-weight) and Medetomidine (0.04 mg/kg body-weight) both hind legs were shaved. Animals were weighed, intubated and received analgesia with Buprenorphine (0.3 ml i.m.). Perioperative antibiotic prophylaxis with Enrofloxacine s.c. was given immediately before the operation; inhalative narcosis was maintained with isoflurane.

**Figure 1:**
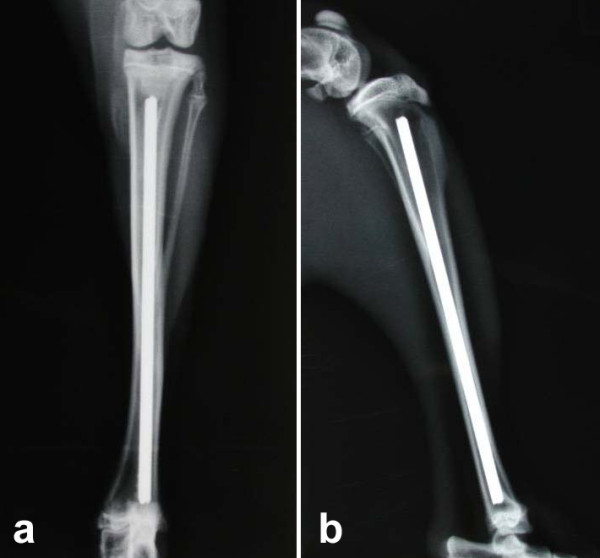
**X-ray of the tibia postoperatively (a) a.p.-view (b) lateral view**.

During the initiation of anaesthesia, the lyophilized COPROG-formula was mixed with the thrombin component of the fibrin glue. The operation was executed under sterile conditions. After incision of the tibia, a hole of 3.2 mm diameter was drilled into the corticalis medial to the tuberosity. Subsequently, the medullary cavity was reamed first with a 2 mm hand brace, followed by 2.5 mm and 2.8 mm. After measuring the length of the tunnel, *Titanium Elastic Nails *(Synthes, Switzerland) of 2.5 mm diameter were cut for later insertion. In all groups except for the blank group, approximately 0.3 ml fibrin glue was injected into the reamed marrow. The fibrinogen component was injected first, followed by the thrombin part, carrying 200 μg of COPROG containing 84 μg of BMP-2 plasmid in the verum group or Luc-Plasmid in the Luciferase group. After that the implant was inserted in anterograde direction (Figure 1). In all animals, both tibiae were operated the same way. After radiographic control of the correct position of the nail, the wound was closed in layers. Postoperative analgesia with Buprenorphine i.m. was given for 2 days.

### Radiographs

Radiographs were taken postoperatively and after the animals were sacrificed, using standardized settings.

### Biomechanical testing and histologic examination

Animals were sacrificed 28 days and 56 days after surgery by intravenous injection of potassium chloride after anaesthesia with Ketamine and Medetomidine. Both tibiae were explanted and randomly assigned for the biomechanical testing or prepared for histomorphometric examination.

**Figure 2:**
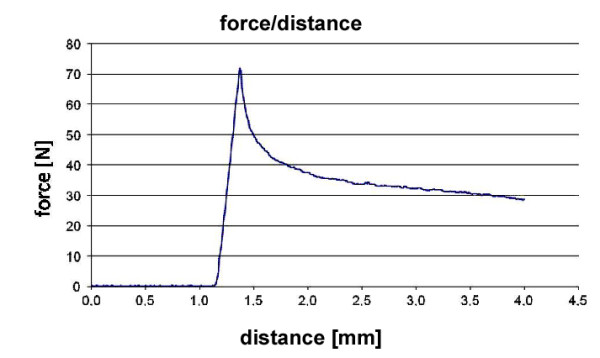
**A typical load-displacement curve with a peak at 72 N, describing the force needed to loosen the nail**.

The biomechanical setup was designed to measure the strength of the attachment of the implant-bone interface. Therefore, we used a push-out device described by Schmidmaier et al. 2002 [22], modified for the bigger rabbit tibia. After cutting off the distal and proximal epiphysis, the bones were prepared carefully in order to reveal about 4 mm of the nail at the distal and proximal end. Subsequently, the tibia was inserted into the testing device and the distal part of the diaphysis was embedded into methyl-metacrylate (MMA). After the cement hardened, the device was positioned into a material testing machine (Zwick, Germany). The machine applied a constant linear anterograde force at a rate of (2 mm/min.) onto the nail and the force was measured and transferred to a computer. The maximum force at ultimate failure was used as parameter for the bone-implant attachment strength. To avoid impreciseness caused by different length of the bones the peak force was set in ratio to the total bone area surrounding the implant. The biomechanical testing resulted in a typical curve with a sharp peak, expressing the force needed to loosen the implant (Figure 2).

The contra-lateral tibiae were prepared for histomorphometrical examination:

After explantation, the proximal and the distal epiphysis were removed to enable the fixation solution (10% normal buffered formaldehyde) to infiltrate into the whole specimen. Specimens were kept in the solution for 5 days followed by dehydration in ethanol of ascending concentrations. Specimens were then embedded in methyl- metacrylate (Technovit 7200, Heraeus-Kulzer, Germany). After polymerization, the resulting blocks including the specimens were cut in longitudinal direction using a cutting device (Exakt, Germany). They were then ground using a grinding device (Exakt, Germany) until the whole specimen could be detected on the surface showing the maximum implant diameter of 2.5 mm. The ground blocks showing the specimens were glued to a microscope slide. The upper parts of the blocks were removed using a diamond band saw (Exakt, Germany), leaving slides of approximately 300 μm of MMA including the specimens. These slides were ground down to 80 μm and staining was performed with Safranin-O and van Kossa. For the histomorphometric analysis, the entire specimen was scanned using a motorized stage with a 10 x objective and a digital camera attached to a microscope (Leica DM-RB, Leica, Germany). The digital pictures were combined with the use of a computer-software (Mosaix, Zeiss, Germany).

**Figure 3:**
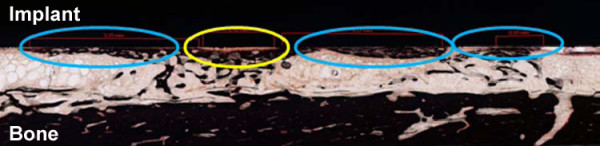
**Histologic preparation stained with Safranin-O/van Kossa**. Blue circles are marking a zone of direct bone contact, the yellow circle marks a zone of indirect bone contact.

To define the bone-implant contact as a sign of integration, the length of all sections where bone was tangent to the implant was measured and set as a ratio to the entire implant length, resulting in a percentage of implant surface covered by bone. The analysis differentiated between direct bone contact, where calcified tissue was directly adjacent to the implant and indirect contact, where bone had grown close to the implant, but a gap was visible (see Figure 3).

### Examination of the Luciferase group

In the Luciferase group, where a plasmid encoding for Luciferase replaced the BMP-2 encoding plasmid, animals were sacrificed at days 4, 7 and 28. Tissue from the operated tibiae, brain, lungs, liver, spleen, testicles and muscle was taken. Also bone samples from the not operated forelegs were analyzed. The bone was grinded with a cooled grinding device before processing. For analysis, the tissue of the parenchymatous organs was homogenized and lysed.

Total RNA was extracted using „RNeasy" Kit^® ^(Quiagen, Germany). Concentration and purity was determined photometrically at 260/280 nm. Approximately 80 ng of total RNA were used for Reverse Transcription PCR. Thereby, single-stranded mRNA was transcribed into complementary DNA (cDNA). In the following non-quantitative PCR the luciferase transcripts were amplified with specific luciferase primers (f 5' ctg aat aca aat cac aga atc gtc g 3'; r 5'aaa tcc ctg gta atc cgt ttt aga 3'). Additionally, the housekeeping gene GAPDH (Glyceraldhyde-3-phosphate-Dehydrogenase) was amplified (f 5'gca tgt cag atc cac aac gga t 3'; r 5'tgt cag caa tgc atc ctg ca 3'). All PCR products were detected on 1.5% agarose gel (Serva) with Ethidiumbromide (Merck, Germany).

### Statistics

Animals were randomized in a blinded manner by drawing lots before the operation. The tibiae (right or left) were also randomized for histological and biomechanical investigation.

To determine statistically significant differences in the histomorphometrical and biomechanical results, a Kruskal Wallis followed by Mann-Whitney Test and Bonferroni Holm correction was used (SPSS 14.0, SPSS Inc., Chicago, USA).

## Results

One animal of the 28 days Luciferase group died during anaesthesia and was excluded from the study. A connection to the vector administration could not be found. All other animals tolerated the procedure well.

Neither the clinical appearance nor the blood specimens suggested an infection at the wound site. In some animals, a transient swelling at the nail insertion site was observed.

### Radiographic Examination

Radiographs showed that the implants were correctly positioned and had a similar fitting in all animals. No dislocations, fractures, or other abnormalities were observed postoperatively or after scarifying. Radiographic analysis did not reveal any difference between the groups at any of the time points.

### Biomechanical testing

**Figure 4:**
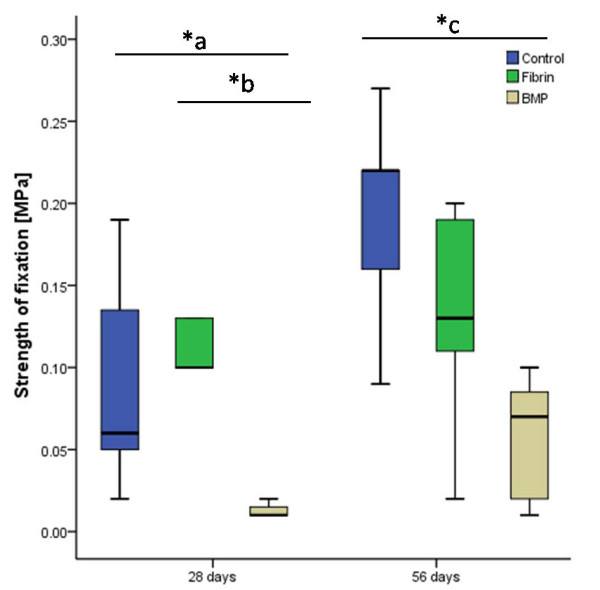
**Shear forces needed to loosen the implant**. Highest results were found in the control group after 56 days. *a p = 0.001 *b p = 0.005 *c p = 0.002. The boxes show the 25th and 75th percentile and the band in the box is the 50th percentile (the median). The whiskers represent the minimum and maximum of all the data.

Similar to the radiographic examination, gross observation revealed no fractures or abnormalities to any of the bones. Throughout the groups, the recording of the testing process showed a typical load-displacement curve with a steep start and a peak, when the force needed to loosen the implant had been reached (Figure 2). The peak force was set in relation to the length of the tested bone to compensate for differences in length between the specimens. In the blank control group an increase in the strength of fixation was detectable between day 28 and 56. This increase in implant fixation over time was less pronounced in the two other groups. The strength of fixation was significantly lower in the verum group at both time points compared to the blank control group. The difference between the blank control group with no filling of the medullar cavity and the fibrin glue group was not significant at either of the time points (Figure 4).

### Histomorphometric Analysis

At day 28 after operation, the measured direct and indirect bone implant-contact was greatest in the blank control group. The fibrin glue group showed almost the same results for direct contact as for indirect contact. The verum group showed significantly lower direct and indirect contact compared to the other groups. These findings support the biomechanical results after 28 days, where the verum group showed significantly lower strength of fixation compared to the other groups.

**Figure 5:**
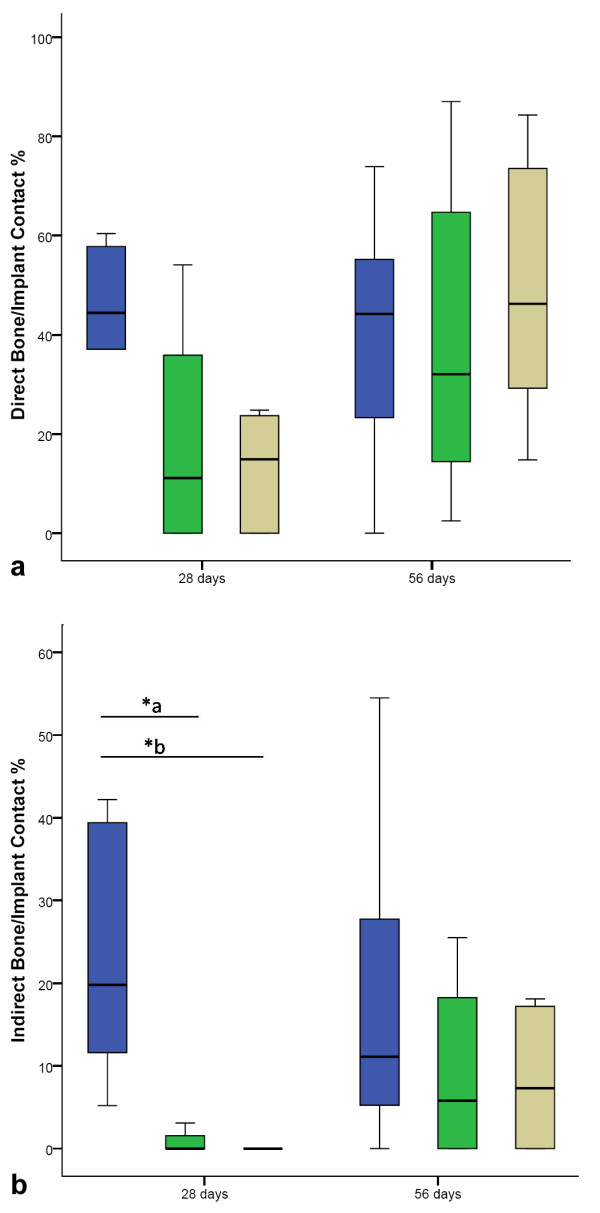
**Direct and indirect bone-implant contact**. a) Direct bone-implant contact: after 28 days significantly less direct contact in the BMP-2 plasmid group. After 56 days the results were comparable in all groups. b) Indirect bone-implant contact. Also, significantly less in the BMP-2 plasmid group after 28 days. After 56 days, again comparable results of all groups. *a p = 0.02 *b p = 0.001. The boxes show the 25th and 75th percentile and the band in the box is the 50th percentile (the median). The whiskers represent the minimum and maximum of all the data.

After 56 days, the results of the verum group were highest regarding the direct bone-implant contact, but did not differ significantly from the blank control group. Indirect contact was comparable in both groups. The fibrin glue group showed the least amount of direct contact after 56 days, while the indirect contact did not differ significantly after 56 days (Figure 5).

### Luciferase group

One animal of the Luciferase group died during anaesthesia.

In 20 of the 22 explanted tibiae Luciferase was detected. In two animals Luciferase could only be found in one of the two tibiae (one of the 4 days group and one of the 28 days group). This means that transfection was successful in 90.9%.

Independent of the time point, luciferase-RNA could be detected after 4, 7 and 28 days. Thus demonstrating a successful transfection had been achieved.

However in the majority of animals, luciferase-RNA was also found in other tissues besides the treated tibiae. There was no obvious relationship between time points and detection of luciferase-RNA, transfection of heterotopic organs took place without any pattern throughout all groups (Table 1).

**Table 1:** Results of the Luciferase mRNA detection in bone and other organs

	Specimen from						operated tibia
**animal**	**brain**	**lung**	**liver**	**spleen**	**testicles**	**muscle**	**left/right**

4d-1	neg.	neg.	neg.	neg.	neg.	neg.	pos./pos.
4d-2	(pos.)	(pos.)	neg.	neg.	(pos.)	neg.	pos./pos.
4d-3	neg.	pos.	neg.	pos.	(pos.)	neg.	neg./pos.
4d-4	pos.	pos.	pos.	(pos.)	pos.	pos.	pos./pos.
7d-1	neg.	neg.	neg.	neg.	(pos.)	neg.	pos./pos.
7d-2	neg.	pos.	neg.	neg.	neg.	neg.	pos./pos.
7d-3	neg.	pos.	(pos.)	(pos.)	(pos.)	neg.	pos./pos.
7d-4	(pos.)	pos.	neg.	pos.	pos.	neg.	pos./pos.
28d-2	neg.	(pos.)	(pos.)	pos.	pos.	neg.	neg./pos.
28d-3	neg.	neg.	(pos.)	neg.	(pos.)	neg.	pos./pos.
28d-4	neg.	neg.	(pos.)	(pos.)	pos.	neg.	pos./pos.
**total: neg.**	**8**	**4**	**6**	**5**	**2**	**10**	**2**
**total: pos.**	**1**	**5**	**1**	**3**	**4**	**1**	**20**
**total: (pos.)**	**2**	**2**	**4**	**3**	**5**	**0**	**0**

Detection of Luciferase mRNA in bone and other organs Number and kind of tissue in animals being positive for Luciferase RNA in heterotopic organs. pos. = positive signal, (pos) = weak signal, neg. = no signal.

## Discussion and Conclusion

Accelerated and improved implant integration could have a significant impact on implant survival, reduction of hospitalization and patient satisfaction. Several authors have described improved bone-implant healing using recombinant BMP-2 or other BMPs [23-25]. Gene therapy offers a promising alternative to the direct application of a recombinant protein by stimulating local target cells to produce more of a desired product, e.g. a growth factor for a period of time that lasts longer than a single application of a recombinant protein. A vector is needed to deliver the genetic information into the target cell. Subsuming the differences between viral vectors and non-viral methods, it is most important that non-viral vectors are unable to match viral vectors concerning their potency in transfecting the target cells. On the other hand, viral vectors are believed to be not as secure, i.e. being more at risk to cause problems due to immunogenicity or mutagenicity, whereas non-viral vectors are believed to be safer. Unlike in systemic genetic disorders where one might want to transfect a majority of target cells of an individual by a systemic application of a gene formula, in the case of tissue repair or regeneration only a local effect is required and desired. A systemic transfection of cells would be an intolerable safety issue. In this study it could be proven that a gene transfer using the non-viral gene vector COPROG was achieved. Transfection could be confirmed by detecting the Luciferase reporter-gene via rtPCR in the operated tibiae. Luciferase RNA could be found in all of the 11 animals included in the reporter-gene groups.

The biomechanical results did not meet the expectations concerning the effect of BMP-2. A significantly weaker implant incorporation was measured at 28 and 56 days after surgery when the BMP-2 plasmid was used. This result corroborates the belief that BMP-2 under certain conditions is also capable of impairing bone formation rather than enhancing it. In recent years, many studies have shown the potency of BMP-2 to promote bone healing. BMP-2 is commonly used clinically to accelerate bone healing after fractures or in cases of non-union as well as in spinal fusion. Some experimental studies showed an improved bone-implant interface through the use of recombinant BMP-2 protein [12,13]. However, there are reports that the effect of BMP-2 can differ fundamentally as the protein is not only capable of enhancing bone formation but also of promoting its degradation by stimulating osteoclasts [26-28]. The circumstances under which the resorption is more pronounced than the bone formation in vivo is not yet clear. In 1996 Jepsson et al. found unexpected inhibitory effects of BMP-2 on bone formation in an established rabbit model. The experiment was repeated in 1999 with variations in dosage, type of BMP and other experimental design modifications. The inhibitory effect was a consistent finding but a mechanism responsible for it could not be determined [29]. Sena et al. found a dosage dependent negative effect of TGF-ß2 on strength of fixation and bone/implant contact in a well established rat model. Interestingly, the bone volume was increased in the TGF-ß2 treated animals [30]. Stadlinger et al. ruled out a detrimental effect of BMP-4 on implant integration in a miniature-pig model when different surface modifications of collagen, collagen and chondroitin sulfate and collagen, chondroitin sulfate and BMP-4 were compared [31]. Liu et al showed in a pig model that different modes of delivery for BMP-2 change the osteoconductivity of surfaces. Different surfaces (metal with/without CaP coating) with or without incorporated or absorbed BMP-2 were investigated. New bone formation was highest in the coated and uncoated groups bearing no BMP-2 followed by the groups where BMP-2 was incorporated in a coated surface. The lowest results concerning deposited bone were found in the coated implants bearing only adsorbed BMP-2. As a second parameter in the study of Liu et al. the interface coverage with bone was examined. This was found highest for blank coated implants, followed by coated implants bearing incorporated or incorporated and adsorbed BMP-2. The lowest results were found in the uncoated implants bearing adsorbed BMP-2. The authors conclude, that osteoconductivity of an implant surface can be significantly influenced not only by BMP-2 but also by the mode of delivery [32]. Egermann et al. found a systemic inhibition of bone formation in a sheep model after local application of adenoviral vectors encoding BMP-2. The effect could be detected by a micro-CT scan 8 weeks after creating standardized defects of the iliac crests on both sides and unilateral local application of Ad.BMP-2. The effect might have been connected to an increased inflammatory response since the histological analysis showed an elevated level of inflammatory cells at the treated bone defects [33]. All these findings and the results of our study are in contrast to the generally accepted positive impact of BMP-2 in fracture healing. Possibly the unexpected effects seen in our present study also can be explained by the different ways new bone is formed during secondary fracture healing and implant integration. While in fracture healing bone formation usually happens via a chondrogenesis and endochondral ossification, unless anatomical repositioning is achieved, implant integration occurs via [34]. BMP-2 is known to promote an endochondral ossification pattern [35], which could interfere with the primary bone-implant healing process and by this account for the poor results of the groups treated with BMP-2 plasmids compared to the other groups.

The histological findings at day 28 supported the biomechanical results. The BMP-2 plasmid group showed the least direct and indirect bone contact. At day 56 the biomechanical results were lowest for the BMP-2 plasmid group while the histomorphometry showed comparable direct bone-implant contact and indirect bone-implant contact in all three groups, not elucidating the biomechanical findings. Further conclusions how the weak implant healing connects to the comparatively large bone-implant contact zone could not be drawn from this experimental design.

In a recent study Sena et al. saw a dose dependent lowering of bone implant contact and fixation strength in a similar model, using a rat femur and different dosages of rhTGFß [30]. In this study, rhTGFb enhanced bone formation at dosages of 5, 10 and 20 micrograms/implant, while at the same time all concentrations of rhTGFß lowered the bone-implant contact and fixation strength. The authors concluded that for fixation strength the location of bone formation is also important, in addition to the amount of bone formation. These findings correlate with our results. Despite a comparable bone-implant contact at day 56, we did not see an improvement in fixation strength.

In the Luciferase group the reporter gene Luciferase could also be found irregularly distributed in heterotopic organs in 10 out of 11 animals. The application of the COPROG/fibrin mixture had been performed with a great deal of caution in order not to spread the formula elsewhere than the wound site. Therefore, the most reasonable explanation for the contamination might be the systemic spread of the locally applied COPROGs due to the rising of the intramedullary pressure during the insertion of the implant. By this, vector loaded fibrin particles might have been pushed into the venous vessels of the tibial bone leading to a systemic distribution of the COPROGS - comparable to the pathogenesis of fat embolism during intramedullary nailing or total joint arthroplasties. Of the systemically transfected organs, muscle tissue showed by far the lowest transfection rate. It could only be found in 1 animal of the 4-day group.

In summary, it could be proven that transfection using the copolymer protected gene vector was achieved, however without a stimulation of implant integration due to the BMP-2 plasmid application. The systemic distribution of the vector, as we found in our reporter gene groups, is not desired and demands further improvement. Fibrin glue as a drug carrier was chosen to release the plasmid formulation due to degradation of the fibrin matrix. This mechanism had been described earlier[20]. The gene-therapeutic approach was used to provide a steady production of BMP-2 at the wound site. Our study supports findings that under certain circumstances BMP-2 might impair implant integration. This work was targeted as a proof of concept study and therefore has limitations in providing information about the exact mechanisms which led to the observed results, e.g. a possible dose-dependent effect of BMP-2. But in this study the proof of transfection in a large animal model using the non-viral vector COPROG could be demonstrated for the first time. The systemic effect means a security risk on the one hand, but shows the capacity of the formula to not only act locally.

## Competing interests

The author declares that they have no competing interests.

## Authors' contributions

BF was the surgeon who carried out the operations. He took part in designing the study and was responsible for the biomechanical testing and the preparation of histological specimens.

BW took part in designing the study and performed the statistical analysis. CH, JH and YF assisted in the surgical procedures and were responsible for animal care. CH mainly contributed to the histological examinations, JH carried out the biomechanical studies and YF was responsible for the PCR assays. CP and AS were in charge of the production of the gene vector. GS designed the study for the most part and was responsible for the coordination between the study groups in Berlin and Munich.

All authors read and approved the final manuscript.

## Pre-publication history

The pre-publication history for this paper can be accessed here:

http://www.biomedcentral.com/1471-2474/12/163/prepub
